# The Mediating Role of Gut Microbiota on the Association Between Dietary Quality and Cancer-Related Fatigue Among Breast Cancer Patients: A Cross-Sectional Study

**DOI:** 10.3390/nu16244371

**Published:** 2024-12-18

**Authors:** Jianyun He, Lan Cheng, Xinxin Cheng, Yuting Wang, Xiaoxia Lin, Shufang Xia

**Affiliations:** Wuxi School of Medicine, Jiangnan University, Wuxi 214122, China; hejianyun@stu.jiangnan.edu.cn (J.H.); chenglan@stu.jiangnan.edu.cn (L.C.); chengxinxin@stu.jiangnan.edu.cn (X.C.); 6232807031@stu.jiangnan.edu.cn (Y.W.); linxiaoxia@stu.jiangnan.edu.cn (X.L.)

**Keywords:** breast cancer, cancer-related fatigue, nutrients, dietary quality, gut microbiota

## Abstract

Objectives: Cancer-related fatigue (CRF) is highly prevalent in patients with breast cancer, resulting in undesirable outcomes and even reduced survival rates. This cross-sectional study investigated the relationship between dietary quality and CRF in patients with breast cancer, and the potential role of gut microbiota (GM) in this association. Methods: Dietary intake and CRF were evaluated in 342 patients, with 64 fecal samples collected for 16sRNA sequencing and 106 plasma samples for tryptophan (TRP) metabolite determination. Results: A total of 149 (43.6%) patients experienced CRF, which was significantly associated with low intakes of protein, vitamin A, vitamin E, dietary fiber, phosphorus, magnesium, potassium, iron, and copper (*p* < 0.05), and a remarkably low Chinese Healthy Eating Index (CHEI) score (*p* < 0.05). CRF patients had decreased GM diversity, an unhealthier GM composition, lower TRP concentrations, and a higher kynurenine (KYN)/TRP ratio (*p* < 0.05). Mediation analyses revealed that both the Sobs index (ACME = −0.0005; 95% CI −0.0051, −0.0001; *p* = 0.034) and the Chao index (ACME = −0.0005; 95% CI −0.0050, −0.0001; *p* = 0.033) were significant mediators of the correlation between total CHEI score and CRF. Conclusions: The presence of CRF in patients with breast cancer might be correlated with inadequate nutrient intake and low dietary quality via GM-dependent pathways.

## 1. Introduction

Breast cancer has the second highest incidence of all cancers and is an important cause of cancer-related deaths in women worldwide [1]. Cancer-related fatigue (CRF), a common, long-lasting, and devastating symptom in breast cancer patients, with a prevalence of 58% to 94% during chemotherapy [2], negatively affects professional work, interpersonal relationships, mood, daily activities, and even survival [3]. Research has demonstrated that participants with persistent CRF had a 2.54 times higher risk of death compared to those without CRF [4]. Unfortunately, CRF is profoundly neglected both by patients and healthcare professionals. It has been reported that 75.0% of Chinese cancer survivors have experienced CRF, but only 10.5% of these patients had heard of CRF, and as few as 4.6% of the patients had taken active actions to intervene with CRF [5]. Although key recommendations on the management of CRF have been proposed in some guidelines [6], no consensus has been reached. Therefore, in order to provide more detailed management measures for the prevention and treatment of CRF, it is critical to recognize the underlying risk factors.

Accumulating evidence indicates that factors contributing to CRF might include sociodemographic, tumor-related, treatment-related, and psychological factors, as well as lifestyle [7]. Diet, one of the factors associated with CRF, can be changed by cancer patients during treatment, leading to improvements in or the exacerbation of CRF. It has been reported that diets rich in protein, dietary fiber, polyunsaturated fatty acids (PUFAs), vegetables, and fruits are negatively correlated with CRF [8,9,10]. Recently, it has been widely recognized that exploring the association between overall dietary quality and CRF may provide more comprehensive dietary recommendations for breast cancer patients experiencing CRF. For example, the Mediterranean dietary pattern [11], anti-inflammatory diets [12], and a high-quality diet with a higher Healthy Eating Index (HEI)-2010 [13] have been suggested to reduce the prevalence of CRF. The Chinese Healthy Eating Index (CHEI), with high reliability and validity in assessing the dietary quality of Chinese residents [14], shows a strong association with cancers [15], diabetes [16], frailty [17], and other diseases. However, no studies exploring the association between the CHEI and CRF in breast cancer patients have been released.

Although the pathophysiology of CRF has not been fully described, the overactivation of the tryptophan (TRP)–kynurenine (KYN) pathway has been reported to be responsible for CRF [18]. TRP, a precursor of the neurotransmitter serotonin (also called 5-hydroxytryptamine—5-HT), cannot be synthesized by the body and must be obtained from food. 5-HT dysregulation is reported to be closely linked to the development of CRF [19]. Therefore, the degradation of TRP via the TRP-KYN pathway reduces the amount of TRP available for normal physiological function. The gut microbiota (GM) possesses regulatory roles in metabolism and psychological function. However, gut dysbiosis can easily occur as a result of the cancer itself or chemotherapy, leading to the dysregulation of TRP metabolism and the serotonergic system [20], and ultimately to CRF. Although a variety of host-endogenous and host-exogenous factors have been shown to influence the GM, diet is a key determinant of the structure and function of the gut microbial community [21], and also provides TRP to produce 5-HT. We speculated that diet might regulate the GM to influence TRP metabolism and ultimately lead to CRF in breast cancer patients.

Indeed, cancer patients often lack adequate dietary health education. A study showed that 62.7% of cancer patients failed to receive any advice to improve their appetite and nutrition [22]. Since CRF is highly prevalent and has significant impacts on quality of life and survival in breast cancer patients, it is urgent to reveal the correlation between dietary quality and CRF, as well as the possible underlying mechanisms involved. Therefore, we performed a cross-sectional study to explore the above issues from the perspective of CHEI-based dietary quality, which might provide guidance for patients in terms of dietary recommendations, thereby improving dietary quality and reducing the risk of CRF in breast cancer patients.

## 2. Materials and Methods

### 2.1. Participants

After obtaining approval from the Medical Ethics Committee of Jiangnan University (JNU20230601IRB06) and registering at the Chinese Clinical Trials Registry (ChiCTR2300074535), we recruited 342 participants at the Affiliated Hospital of Jiangnan University from August 2023 to April 2024. The inclusion criteria for participants included the following: (1) female breast cancer patients without metastasis or diffusion; (2) hospitalized for postoperative chemotherapy; (3) age of 18 years or older; (4) normal cognitive function and reading ability; (5) voluntary participation. The exclusion criteria included the following: (1) previous diagnosis of mental illness; (2) co-existence with other cancers; (3) history of digestive-system-related disorders; (4) being pregnant or lactating; (5) utilization of radiation or chemotherapy drugs, antibiotics, probiotics, prebiotics, steroids, or immune-suppressant agents within 21 days; (6) suffering from serious comorbidities such as heart disease; (7) other reasons considered by the researcher to render patients unsuitable for participation in the study. We obtained written informed consent from all participants.

### 2.2. Sample Size

We used the following formula to calculate the sample size:

$$n=\frac{Z_{\alpha /2}^{2}\left(1-p\right)p}{\delta^{2}}$$

in which *α* = 0.05 and *Z_α_*_/2_ = 1.96. *P*, the prevalence of CRF among breast cancer patients, was reported as 0.497 in one study [23], with the permissible error δ = 0.15, *p* = 0.07455. Accordingly, the minimum sample size was 173.

### 2.3. Assessment of CRF

CRF was assessed with the International Classification of Diseases, 10th Revision (ICD-10) (Table S1) [24]. According to ESMO guidelines, ICD-10 diagnostic criteria are currently the “gold standard” for diagnosing CRF [6]. Six of the eleven criteria and self-reported functional limitations are prerequisites for diagnosis. We asked the participants who felt fatigued at least a few days per month during treatment about the effects of fatigue on their daily functioning, including emotional, physical, social, behavioral, economic, and occupational factors. We also asked the patients about their current level of fatigue in addition to how they coped with fatigue during and after treatment. The detailed diagnostic interview guide for CRF is shown in Table S2 [25].

### 2.4. Dietary Intake Assessment

The 3-day, 24 h dietary recall interview was conducted after the patient was admitted to the hospital. With the help of food models and atlases, each newly hospitalized participant was asked to describe the foods consumed in a 24 h period and their cooking methods through face-to-face interviews. After the participants were discharged from the hospital, we collected the remaining two 24 h dietary recalls via WeChat. After the chemotherapy-induced gastrointestinal disorders of the patients had disappeared during their stay at home, the participants or their caregivers weighed and photographed the food, cooking oils, and condiments consumed and sent the photos to the researchers, followed by confirmation on the amounts of food via video calls. The collected data were used to obtain daily nutrient intake using the Nutrition Calculator v2.8.0.5 (Beijing, China), and the mean nutrient intake of 3 days was used for subsequent statistical analysis.

### 2.5. Calculation of CHEI

The CHEI is derived from 17 components, including 12 adequacy components (foods that are often considered to be good for health, such as whole grains, vegetables, and fruits) and 5 moderation components (foods that are considered harmful to health when consumed in excess, e.g., red meat and cooking oils) [26]. Each component of the CHEI is scored based on the corresponding food component intake per 1000 calories. The total CHEI score is the sum of the 17 component scores, where the score for each component ranges from 0 to 5, and the scores for the three components of fruits, edible oils, and sodium range from 0 to 10. Accordingly, total CHEI score ranges from 0 to 100. A higher score on the CHEI indicates better dietary quality.

### 2.6. Plasma KYN/TRP Ratio Measurement

We retained fasting blood samples after obtaining informed consent from the patients and collected a total of 106 samples, of which 34 were from CRF patients and 72 from non-CRF (NCRF) patients. Plasma was extracted by centrifugation at 3500 rpm for 10 min. Plasma TRP (catalog MM-51157H1 and MM-51157H2) and KYN (catalog MM51191H1 and MM51191H2) levels were tested with ELISA kits (Jiangsu Meimian Industrial Co., Ltd., Yancheng, China).

### 2.7. GM Analysis

In total, 64 fecal samples (25 CRF samples and 39 NCRF samples) were collected before the initiation of chemotherapy. DNA was extracted with an E.Z.N.A.^®^ Soil DNA Kit (Omega Bio-Tek, Norcross, GA, USA), and then the concentration and purity were determined. PCR amplification of purified DNA with the hyper-variable V3-V4 region of 16S rRNA was conducted using universal primers with an ABI GeneAmp^®^ 9700 PCR thermocycler (ABI, Carlsbad, CA, USA), followed by the establishment of sequencing libraries. The Illumina MiSeq PE300 platform (Illumina, San Diego, CA, USA) was used to sequence the library. The sequencing reads were demultiplexed, quality-filtered using fastp version 0.20.0, and merged with FLASH version 1.2.7.

Operational taxonomic units (OTUs) were clustered based on a 97% similarity of valid sequences using the UPARSE method. GM diversity was evaluated using the Chao index, Shannon index, Sobs index, and Simpson index. To assess microbial community differences, beta diversity was assessed using a principal coordinate analysis (PCoA) of weighted UniFrac distances, and statistical significance was analyzed using an analysis of similarity (ANOSIM). Differences in the composition of the GM (*p*-values were corrected for multiple testing using the Benjamini–Hochberg false discovery rate (FDR) method) and the alpha diversity of bacterial communities between CRF and NCRF patients were analyzed using the Wilcoxon rank-sum test. The linear discriminatory analysis effect size (LEfSe) analysis method with default criteria (*p* < 0.05 with a non-parametric factorial Kruskal–Wallis rank-sum test and linear discriminant analysis (LDA) score > 4) was used to identify the primary contributing bacteria, as well as the differences in the samples from the CRF and NCRF groups, respectively. All data analyses were performed on the Majorbio Cloud Platform (www.majorbio.com, accessed on 8 June 2024).

### 2.8. Assessment of Other Variables

Sleep disorders, pain, anxiety and depression status, and physical activity were assessed as described in the Supplementary Materials.

### 2.9. Statistical Analyses

We performed statistical analyses with SPSS 26.0 (IBM SPSS Inc., Chicago, IL, USA). The Kolmogorov–Smirnov test was used for the evaluation of normality. Normally distributed variables are shown as mean ± SD, and non-normally distributed variables are shown as median (25th, 75th percentile). Then, an independent samples *t*-test or the Mann–Whitney U test were performed to assess the differences in the variables between the CRF and NCRF groups. Categorical variables are presented as frequencies and percentages (*n*, %) and analyzed by Chi-squared test. Multiple logistic regression analysis was used to analyze the correlation between dietary intake, total CHEI score and its components, or the GM and the occurrence of CRF. Linear regression analysis was used to explore the correlation between nutrients, the CHEI, and the GM. R Mediation package 4.5.0 was used to determine the mediating effect of the GM on the association between total CHEI score or dietary nutrients and CRF, and the average causal mediation effect (ACME), the average direct effect (ADE), and the average total effect (ATE) were estimated. Non-parametric bootstrapping with 5000 replications was used to estimate the 95% confidence interval (CI). All analyses were adjusted for age, BMI, visual analogue scale (VAS) score, family monthly income, sleep disorders, anxiety, depression, and energy. Since energy was corrected for in the calculation of the CHEI, only the above covariates other than energy were corrected for when analyzing the association between CHEI and CRF. *p* < 0.05 was considered statistically significant.

A total of 263 variables were analyzed, and 77 were found to be significant with a *p*-value < 0.05. After Bonferroni’s correction, the reference *p*-value should drop to 0.00019 (0.05/263) to exclude variables that appear significant by chance. But based on this adjusted reference *p*-value, there were no *p*-values that remained significant. However, under the null hypothesis that there was no association, we would have expected less than 14 *p*-values (5% of 263) to be <0.05. Instead, we observed that 29% (77 out 263) of the *p*-values were <0.05, suggesting the presence of actual associations in our data [27]. For this reason, *p*-value < 0.05 was kept as threshold for statistical significance in all analyses.

## 3. Results

### 3.1. Participants’ Characteristics

The characteristics of the CRF and NCRF groups are presented in Table 1. Out of the 342 participants, 149 (43.6%) patients experienced CRF. Statistical differences between the CRF and NCRF groups were found in age (*p* = 0.045), VAS score (*p* = 0.005), marital status (*p* = 0.025), education level (*p* = 0.027), and family monthly income (*p* < 0.001). The CRF group had a significantly higher percentage of patients with sleep disorders, anxiety, and depression (*p* < 0.001) than the NCRF group.

### 3.2. Nutrient Intake and CHEI Scores

Table 2 presents the nutrient intakes of breast cancer patients with CRF and NCRF. CRF participants consumed remarkably lower amounts of energy, macronutrients (fat, protein, and carbohydrates), dietary fiber, vitamins (A, E, B1, B2, B6, and C, folate, biotin, and niacin), minerals (such as calcium, sodium, and iron), choline, fatty acids (including PUFA, omega-3, and omega-6 PUFAs), and TRP than NCRF patients (*p* < 0.05). Additionally, patients with CRF had significantly lower total CHEI scores and component scores (including scores for soybeans, dark vegetables, whole grains and mixed beans, and seeds and nuts) than NCRF patients (Table 3, *p* < 0.05).

### 3.3. Association Between Nutrients, CHEI Score or Its Component Score, and the Occurrence of CRF in Breast Cancer Patients

After adjustment for age, BMI, VAS score, family income, sleep disorders, anxiety, depression, and energy, protein (OR = 0.98; 95% CI 0.97, 0.99; *p* = 0.016), dietary fiber (OR = 0.94; 95% CI 0.88, 0.99; *p* = 0.024), vitamin A (OR = 0.99; 95% CI 0.99, 1.00; *p* = 0.006), vitamin E (OR = 0.94; 95% CI 0.90, 0.98; *p* = 0.003), phosphorus (OR = 0.99; 95% CI 0.99, 1.00; *p* = 0.021), potassium (OR = 0.99; 95% CI 0.99, 1.00; *p* = 0.018), magnesium (OR = 0.99; 95% CI 0.99, 1.00; *p* = 0.008), iron (OR = 0.95; 95% CI 0.90, 0.99; *p* = 0.039), and copper (OR = 0.75; 95% CI 0.60, 0.95; *p* = 0.018) showed significant association with the occurrence of CRF in patients with breast cancer (Table 4). Additionally, both total CHEI score (OR = 0.95; 95% CI 0.93, 0.98; *p* = 0.002) and whole grains and mixed beans (OR = 0.85; 95% CI 0.75, 0.96; *p* = 0.010) were significantly linked with the occurrence of CRF in breast cancer patients (Table 5).

### 3.4. Plasma TRP and KYN Levels

Table 6 shows that although there was no significant difference in plasma KYN levels (*p* > 0.05), plasma TRP levels were significantly lower in CRF patients compared with NCRF patients, and the KYN/TRP ratio was remarkably increased (*p* < 0.05).

### 3.5. GM Diversity

**Figure 1:**
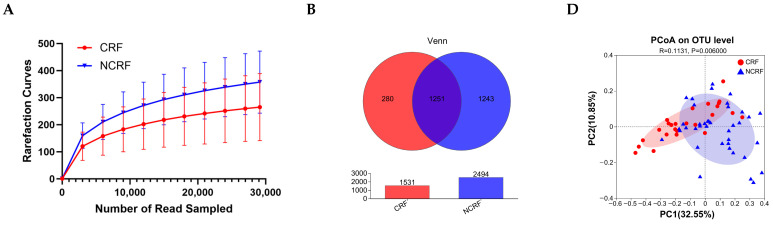

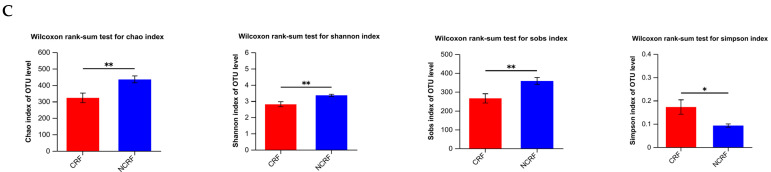
Gut microbiota structure in breast cancer patients with NCRF and CRF. (**A**) Rarefaction curves. (**B**) Venn diagram displaying the shared number of operational taxonomic units (OTUs). (**C**) Chao index, Shannon index, Sobs index, and Simpson index. The Wilcoxon rank-sum test was used. (**D**) Weighted UniFrac distance-based principal coordinate analysis (PCoA). The statistical significance was assessed with analysis of similarities (ANOSIM). CRF, cancer-related fatigue (*n* = 25); NCRF, non-cancer-related fatigue (*n* = 39). * *p* < 0.05, ** *p* < 0.01.

Figure 1A shows that the sequencing depth was enough to explore GM diversity. The Venn diagram demonstrates that there were 1251 OTUs shared by the CRF and NCRF patients (Figure 1B). The results from the alpha diversity analysis (Figure 1C) show that the Chao index (*p* = 0.004), Shannon index (*p* = 0.004), and Sobs index (*p* = 0.005) were remarkably decreased in the CRF patients compared with the NCRF patients, whereas the Simpson index was increased (*p* = 0.013), indicating that CRF patients had decreased GM diversity. The PCoA diagram shows that the GM of the CRF patients remarkably differed from that of the NCRF patients in terms of weighted UniFrac distances (*p* = 0.006, Figure 1D).

### 3.6. GM Composition

**Figure 2:**
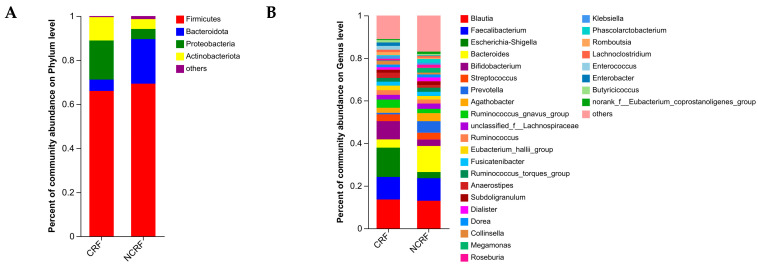
Gut microbiota composition in breast cancer patients with NCRF and CRF. The community structures at the phylum (**A**) and genus (**B**) levels. CRF, cancer-related fatigue (*n* = 25); NCRF, non-cancer-related fatigue (*n* = 39).

**Figure 3:**
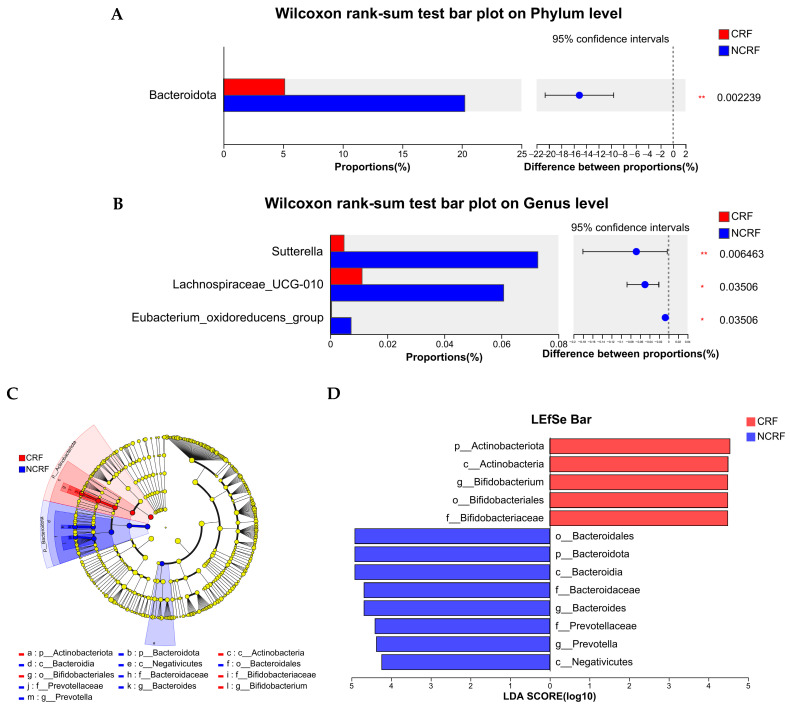
Differentiated microbes between breast cancer patients with CRF and NCRF. Differentiated microbes at the phylum (**A**) and genus (**B**) levels. (**C**) Linear discriminatory analysis effect size (LEfSe) was used to distinguish the differential microbes between the CRF and NCRF patients. (**D**) Linear discriminant analysis (LDA) was performed, and only the microbiota with LDA scores of >4 are shown. CRF, cancer-related fatigue (*n* = 25); NCRF, non-cancer-related fatigue (*n* = 39). * *p* < 0.05, ** *p* < 0.01.

As shown in Figure 2A, in CRF patients, the top two dominant phyla were Firmicutes and Proteobacteria, with abundances of 66.02% and 17.74%, respectively, whereas in NCRF patients, the top two dominant phyla were Firmicutes and Bacteroidota, with abundances of 69.31% and 20.24%, respectively. The common dominant genera (>5%) in CRF and NCRF patients were *Blautia* and *Faecalibacterium*, and *Escherichia-Shigella* and *Bifidobacterium* were dominant genera only in CRF patients, whereas *Bacteroides* and *Prevotella* were dominant genera only in NCRF patients (Figure 2B). Moreover, after FDR correction, the CRF patients demonstrated significantly decreased relative abundances of Bacteroidota (corrected *p*-value = 0.002), *Sutterella* (corrected *p*-value = 0.006), *Lachnospiraceae_UCG-010* (corrected *p*-value = 0.035), and *Eubacterium_oxidoreducens_group* (corrected *p*-value = 0.035), compared with the NCRF patients (Figure 3A,B).

LEfSe analysis showed 13 differentiated taxa with an LDA score > 4.0 from phylum to genus (Figure 3C,D), with p_Actinobacteriota (*p* = 0.022), c_Actinobacteria (*p* = 0.003), *g_Bifidobacterium* (*p* = 0.009), o_Bifidobacteriales (*p* = 0.010), and f_Bifidobacteriaceae (*p* = 0.010) being more abundant in CRF patients, and o_Bacteroidales (*p* < 0.001), p_Bacteroidota (*p* < 0.001), c_Bacteroidia (*p* < 0.001), *g_Bacteroides* (*p* = 0.006), f_Bacteroidaceae (*p* = 0.006), f_Prevotellaceae (*p* = 0.001), *g_Prevotella* (*p* = 0.001), and c_Negativicutes (*p* = 0.010) being more abundant in NCRF patients.

### 3.7. Association Between GM and the Occurrence of CRF or Dietary Intake

After adjusting for age, BMI, VAS score, family monthly income, sleep disorders, anxiety, depression, and energy, the Sobs index (OR = 0.99; 95% CI 0.98, 0.99; *p* = 0.002, Table S3), Chao index (OR = 0.99; 95% CI 0.98, 0.99; *p* = 0.002), and Shannon index (OR = 0.11; 95% CI 0.02, 0.65; *p* = 0.015), PC1 (OR = 0.90; 95% CI 0.84, 0.96; *p* = 0.002), p_Actinobacteriota (OR = 1.01; 95% CI 1.00, 1.02; *p* = 0.013), p_Bacteroidota (OR = 0.98; 95% CI 0.97, 0.99; *p* = 0.004), p_Proteobacteria (OR = 1.01; 95% CI 1.00, 1.01; *p* = 0.042), p_Desulfobacterota (OR = 0.99; 95% CI 0.98, 0.99; *p* = 0.013), p_unclassified_k_norank_d_Bacteria (OR = 0.98; 95% CI 0.97, 0.99; *p* = 0.035), *g_Bacteroides* (OR = 0.99; 95%CI 0.98, 0.99; *p* = 0.013), and *g_Bifidobacterium* (OR = 1.01; 95% CI 1.00, 1.02; *p* = 0.010) were all remarkably correlated with the occurrence of CRF. The association between GM and dietary intake is shown in Table S4. Total CHEI score showed positive associations with the Sobs index (β = 0.39; 95% CI 2.12, 11.08; *p* = 0.005), Chao index (β = 0.37; 95% CI 2.03, 12.55; *p* = 0.007), and Shannon index (β = 0.42; 95% CI 0.02, 0.06; *p* = 0.001), PC1 (β = 0.27; 95% CI 0.07, 1.50; *p* = 0.033), Bacteroidota (β = 0.34; 95% CI 1.66, 12.45; *p* = 0.011), and unclassified_k_norank_d_Bacteria (β = 0.43; 95% CI 1.43, 5.82; *p* = 0.002). Vitamin A demonstrated positive associations with the Sobs index (β = 0.28; 95% CI 0.01, 0.17; *p* = 0.047), the Shannon index (β = 0.27; 95% CI 0.00, 0.01; *p* = 0.033), and Desulfobacterota (β = 0.36; 95% CI 0.07, 0.45; *p* = 0.008). Dietary phosphorus showed a positive association with Desulfobacterota (β = 0.40; 95% CI 0.05, 0.67; *p* = 0.025), while copper had an inverse correlation with Proteobacteria (β = −0.26; 95% CI −83.43, −0.52; *p* = 0.047).

### 3.8. Association Between Total CHEI Score or Nutrients and the Occurrence of CRF with GM as a Mediator

Table 7 shows that the correlation between total CHEI score and the occurrence of CRF was significantly mediated by alpha diversity, as indicated by both the Sobs index (ACME = −0.0005; 95% CI −0.0051, −0.0001; *p* = 0.034) and the Chao index (ACME = −0.0005; 95% CI −0.0050, −0.0001; *p* = 0.033), whereas no significant mediating effects were found for vitamin A, phosphorus, or copper.

## 4. Discussion

This study reveals that breast cancer patients with a poorer dietary quality, as suggested by lower total CHEI scores, had a higher prevalence of CRF. The CRF patients had an insufficient intake of nutrients, including dietary fiber, protein, vitamin A, vitamin E, phosphorus, potassium, magnesium, iron, and copper, and lower whole grain and mixed beans consumption. They also exhibited lower plasma TRP levels and a higher KYN/TRP ratio, less microbial diversity, and an unhealthier GM composition. Mediation analysis demonstrated that alpha diversity mediated the correlation between total CHEI score-based dietary quality and the occurrence of CRF in breast cancer patients.

CRF is a common and distressing symptom in breast cancer patients, leading to numerous adverse outcomes and severely impacting quality of life [7]. Studies have shown that compared to symptoms such as pain, insomnia, and nausea/vomiting, CRF exerts a more substantial negative effect on quality of life [28]. We found that the prevalence of CRF among breast cancer patients was 43.6%, similar to the 46.3% reported in previous research [29]. The effect of age on CRF remains inconclusive. Although it has been suggested that age is not associated with the prevalence of CRF [23], when patient analyses were confined to those with breast cancer, 12 studies reported that younger patients were more likely to experience CRF, whereas only 2 studies suggested that older patients were more susceptible to develop CRF [7]. Our findings showed that the CRF patients were significantly older than the NCRF group. In this study, widowed/divorced/single patients exhibited a higher prevalence of CRF than married patients, which might be due to the fact that married patients could receive more comprehensive life care and psychological support from their spouses to help them better cope with the stresses of illness and treatment. Additionally, both lower education and income have been proven to increase CRF in breast cancer survivors [30,31]; we also observed a higher proportion of CRF patients with lower education and lower family monthly income. This might be due to the patients’ fear of the financial pressures associated with the treatment of the disease, as well as a lack of knowledge about the disease. Consequently, healthcare professionals should focus on breast cancer patients who are older, less educated, have lower family monthly income, and have no spouse to identify potential CRF high-risk groups.

Cancer and its treatment can lead to diet-related disorders, such as altered taste, nausea, vomiting, and anorexia, which result in reduced nutritional intake in cancer patients [32]. Lots of studies have explored the correlation between CRF and single nutrients. In this regard, proteins are beneficial for maintaining or increasing lean body mass, and thus play an important role in CRF [10]. Although no standardized criteria have been established for protein requirements in cancer patients, it is highly likely that they have increased protein requirements compared to their healthy counterparts due to higher rates of protein turnover in the body and increased protein synthesis in the acute phase [33]. A prospective observational study showed that cancer patients receiving chemotherapy with a recent protein intake of less than 1 g/kg body weight had the highest odds of developing CRF [10]. In our study, the daily protein intake was 0.97 g/kg for CRF patients and 1.19 g/kg for NCRF patients, respectively, suggesting that low protein intake might be a contributor to CRF in breast cancer patients. A higher intake of dietary fiber has also been shown to reduce fatigue. In breast cancer survivors, when they consumed less than 25 g of dietary fiber per day, they felt significantly more fatigue on average than survivors who consumed more than 25 g of dietary fiber [34]. Cancer survivors are recommended by the WCRF/AICR to follow the guideline of consuming at least 30 g of dietary fiber every day [35]. Although our results support the notion that dietary fiber is a protective factor for CRF, the median daily dietary fiber intake of the CRF and NCRF patients was only 7.5 g and 9.7 g, respectively, which are both far below the recommended intake. Accordingly, breast cancer patients should be encouraged to increase their intake of dietary fiber-rich foods to prevent the development and progression of CRF. Additionally, several micronutrients have been suggested to have a role in CRF [36]. The Geisinger Rural Aging Study found that individuals reporting higher levels of physical fatigue had lower intakes of vitamin A, magnesium, zinc, and phosphorous compared to those with lower levels of physical fatigue [37]. A prospective, randomized, controlled study demonstrated that the combined use of pentoxifylline and vitamin E decreased fatigue in head and neck cancer patients [38]. We also found an association between vitamin A, vitamin E, phosphorus, potassium, magnesium, iron, copper, and CRF in breast cancer patients. Therefore, supplementing CRF cancer survivors with efficient and safe nutrients such as protein, dietary fiber, vitamins, and minerals may be a beneficial treatment option.

Recently, research on the effects of diet on CRF has shifted from a focus on single nutrients or food components to a broader range of dietary patterns or dietary qualities to more fully understand the effects of diet on CRF. Different dietary patterns, for example, the Mediterranean diet, might decrease the prevalence of CRF [11]. The HEAL cohort study showed that diet quality, as evaluated by HEI-2010, was negatively and independently correlated with fatigue in breast cancer survivors [13]. In a feasibility pilot study, a remote dietary intervention program designed to reduce fatigue over a 12-week period also improved dietary quality and fatigue, and increased whole grain intake in lymphoma survivors [39]. The CHEI is constructed to evaluate the dietary quality of Chinese residents. As a continuous scoring method, it is easy to interpret and useful in its application in different statistical analyses [14]. In our study, decreased CHEI scores indicated poor dietary quality in CRF patients, and after adjusting for covariates, CHEI scores were significantly negatively correlated with CRF. These findings suggest that dietary quality is a crucial factor leading to CRF in breast cancer patients. Therefore, when providing health education to breast cancer patients, healthcare professionals should emphasize to patients the importance of not only ensuring an adequate intake of single nutrients in the diet, but also focusing on an overall balanced diet and improving dietary quality to prevent CRF.

Accumulated evidence suggests that alterations in the GM might be involved in the pathogenesis of fatigue [40]. Although the exact mechanism linking diet and CRF is largely unknown, the GM, a key modulator of physiological and psychological functioning, is susceptible to disruption by cancer progression or chemotherapy and is regulated by diet [41]. We have previously demonstrated that total CHEI score shows positive correlations with the Chao index and the Shannon index, and negative correlation with the Simpson index in breast cancer patients [42]. Meanwhile, the GM has been proven to directly or indirectly control the three TRP metabolic pathways, resulting in the production of 5-HT, KYN, and indole derivatives [43], and thus may lead to CRF [44]. Although there was no direct evidence of a causal relationship between the GM and TRP metabolism in breast cancer patients in this study, several studies have examined the associations between the GM, TRP metabolites, and CRF. In head and neck cancer patients, alpha diversity and the relative abundance of short-chain fatty acid (SCFA)-producing taxa have been reported to be lower in patients with high CRF, whereas the abundance of taxa associated with inflammation was higher [44]. In breast cancer survivors, correlations also exist between alterations in GM composition (e.g., *Faecalibacterium*, *Prevotella*, and *Bacteroides*) and longitudinal changes in fatigue [45]. In our study, mediation analyses indicated that gut microbial alpha diversity (Sobs index and Chao index) significantly mediated the association between dietary quality and CRF in breast cancer patients. Furthermore, CRF patients had a lower abundance of Bacteroidota than NCRF patients, which is contrary to the findings that fatigued rectal cancer patients had a higher abundance of Bacteroidota at the phylum and genera levels than participants without fatigue at the end of chemoradiotherapy [46]. We speculated that the reason for this discrepancy might be related to different cancer types, chemotherapy regimens, and cycles. Considering the fact that Bacteroidota can produce butyrate and SCFA and modulate immunity and anti-inflammation [47], which might be beneficial for CRF treatment, we speculated that increasing the relative abundance of Bacteroidota might be beneficial for CRF. Bifidobacterium belonging to the phylum Actinobacteria also produce butyrate through the fermentation of dietary fiber and contribute to gut homeostasis and host health [48]. Surprisingly, both Actinobacteria and Bifidobacterium were more abundant in the CRF group according to the LEfSe analysis. Our previous study on lung cancer patients also showed a higher abundance of Actinobacteria in frail participants compared to non-frail participants [49]. In the present study, we also found that after FDR correction, the relative abundance of *Sutterella*, *Lachnospiraceae_UCG-010,* and *Eubacterium_oxidoreducens_group* was lower in CRF patients than in NCRF patients. *Lachnospiraceae_UCG-010* belongs to the family of *Lachnospiraceae* and *Eubacterium_oxidoreducens_group* belongs to the family of Eubacterium, both of which had lower abundances within the GM of patients with fibromyalgia syndrome, which is usually associated with chronic fatigue [50]. It has been reported that *Lachnospiraceae* and Eubacterium are key taxa involved in butyrate production, and their reduced abundance might lead to an impaired production of SCFAs, which in turn triggers inflammation [47], suggesting that CRF patients might be in a pro-inflammatory state. However, we also found that *Prevotella* abundance was lower in the CRF group, which contradicts the perception that this bacterium promotes chronic inflammation [51]. The reason for this inconsistency might be that while *Prevotella* thrives in a pro-inflammatory environment [52], disease-related inflammation might directly drive decreased *Prevotella* abundance by creating a microenvironment not suitable for survival [51]. Guided by basic research on neuro-immune interactions, a growing body of research has examined the hypothesis that CRF is driven by the activation of the pro-inflammatory cytokine network [53]. However, the interconnection between the GM and inflammation was not explored in the present research. These results suggest that the GM, as a large assemblage of bacteria, has a complex role in the association between dietary quality and CRF, and further research is still needed. Additionally, the associations between TRP metabolism and CRF have also been extensively studied. Lower plasma TRP contents and increased KYN/TRP ratios have been reported in CRF patients, both in lymphoma survivors [54] and in lung cancer patients [19]. Our research also revealed that CRF patients exhibited decreased plasma TRP, along with an increased KYN/TRP ratio, furthering supporting the role of TRP metabolism in CRF. As a precursor, TRP is metabolically transformed to bioactive metabolites, including 5-HT and KYN. Due to the characteristics of malignant tumors and the use of some chemotherapy drugs, lower 5-HT levels in cancer patients could induce fatigue, depression, malnutrition, and other comorbidities. Dietary intervention with TRP-rich whey protein isolates could inhibit the conversion of TRP to KYN and increase 5-HT production, as well as attenuate depressive-like behavior in mouse models of breast cancer [55]. A community-based study in Bangladesh showed that although the plasma KYN/TRP ratio was not associated with undernutrition in adults, it was reduced after nutrition intervention [56]. Unfortunately, as some patients were reluctant to provide blood samples and stool samples, which needed to be collected before chemotherapy and after admission, the sample size was limited, and there was little overlap between participants who provided both samples. Consequently, we could not analyze the correlation between GM and TRP. More in-depth studies are necessary to explore the pathways and key strains of the GM that contribute to CRF, as well as the causal relationship between diet, the GM, TRP metabolism, and CRF.

In this study, some limitations should be acknowledged. First, dietary intake was assessed by dietary recall. Although we used food models and atlases to help participants recall their intake, recall bias may still exist. Second, only 109 participants agreed to provide blood samples and only 64 participants provided fecal samples for 16sRNA sequencing before chemotherapy, resulting in a much smaller sample size for blood and fecal analyses compared with the questionnaire data. Third, it was not possible to conclude the temporal order of or causality among the CHEI, the GM, and CRF due to the cross-sectional design of the study, which might lead to biased estimates of mediating effects. Future prospective studies could be conducted to investigate the trajectory of CRF and to determine the causal relationships among these factors.

## 5. Conclusions

Our research has illustrated that the high prevalence of CRF in breast cancer patients, with 43.6% of patients experiencing CRF, is possibly associated with a decreased intake of protein, vitamin A, vitamin E, dietary fiber, phosphorus, potassium, magnesium, iron, and copper, as well as poorer dietary quality and lower whole grain and mixed beans intake. The GM of CRF patients was also unhealthy, as evidenced by lower microbial diversity and imbalanced microbial composition, and the Sobs index and the Chao index were mediators in the association between CHEI-based dietary quality and the occurrence of CRF. Healthcare professionals should provide nutritional education to patients, encourage them to consume a balanced diet with adequate nutrients, and ensure dietary quality to maintain GM homeostasis to cope with CRF.

## Figures and Tables

**Table 1:** Characteristics of the breast cancer patients with NCRF and CRF.

Variables	NCRF (*n* = 193)	CRF (*n* = 149)	*p*
Age (years) ^a^	53.2 ± 10.7	55.6 ± 11.1	0.045
BMI (kg/m^2^) ^b^	23.8 (21.9, 25.6)	24.2 (22.1, 26.4)	0.424
VAS score ^b^	0.0 (0.0, 1.0)	0.0 (0.0, 2.0)	0.005
Menopausal status, *n* (%) ^c^			
Pre-menopausal	74 (38.3)	48 (32.2)	0.241
Post-menopausal	119 (61.7)	101 (67.8)	
Marital status, *n* (%) ^c^			
Widowed/divorced/single	3 (1.6)	9 (6.0)	0.025
Married	190 (98.4)	140 (94.0)	
Education level, *n* (%) ^c^			
Primary school or lower	38 (19.7)	35 (23.5)	0.027
Middle school	69 (35.8)	45 (30.2)	
High school/secondary school	33 (17.1)	42 (28.2)	
Junior college or higher	53 (27.4)	27 (18.1)	
Employment, *n* (%) ^c^			
Employed	52 (26.9)	38 (25.5)	0.515
Unemployed	32 (16.6)	32 (21.5)	
Retired	109 (56.5)	79 (53.0)	
Residence, *n* (%) ^c^			
Rural areas	22 (11.4)	28 (18.8)	0.132
Towns	24 (12.4)	14 (9.4)	
Urban areas	147 (76.2)	107 (71.8)	
Family monthly income (RMB), *n* (%) ^c^			
<3000	13 (6.7)	23 (15.4)	< 0.001
3000–5000	68 (35.2)	72 (48.3)	
>5000	112 (58.1)	54 (36.3)	
Family history of cancer, *n* (%) ^c^			
No	177 (91.7)	130 (87.2)	0.177
Yes	16 (8.3)	19 (12.8)	
Presence of comorbidities, *n* (%) ^c^			
No	128 (66.3)	97 (65.1)	0.813
Yes	65 (33.7)	52 (34.9)	
Smoking status, *n* (%) ^d^			
Never	183 (94.8)	139 (93.3)	0.550
Former/current	10 (5.2)	10 (6.7)	
Physical activity level, *n* (%) ^c^			
Low	40 (20.7)	42 (28.2)	0.172
Moderate	143 (74.1)	103 (69.1)	
High	10 (5.2)	4 (2.7)	
Sleep disorders, *n* (%) ^c^			
No	146 (75.6)	67 (45.0)	< 0.001
Yes	47 (24.4)	82 (55.0)	
Anxiety, *n* (%) ^c^			
No	177 (91.7)	101 (67.8)	< 0.001
Yes	16 (8.3)	48 (32.2)	
Depression, *n* (%) ^c^			
No	179 (92.7)	104 (69.8)	< 0.001
Yes	14 (7.3)	45 (30.2)	
Location of diseases, *n* (%) ^e^			
Left breast cancer	91 (47.2)	75 (50.3)	0.557
Right breast cancer	96 (49.7)	72 (48.3)	
Double breast cancer	6 (3.1)	2 (1.4)	
Cancer stage, *n* (%) ^c^			
I	69 (35.7)	44 (29.5)	0.319
II	108 (56.0)	87 (58.4)	
III	16 (8.3)	18 (12.1)	
Type of surgery, *n* (%) ^c^			
Mastectomy	118 (61.1)	99 (66.4)	0.313
Lumpectomy	75 (38.9)	50 (33.6)	
Number of chemotherapy cycles ^b^	2.0 (2.0, 4.0)	2.0 (1.0, 4.0)	0.936
Presence of anemia, *n* (%) ^c^			
No	151 (78.2)	118 (79.2)	0.831
Yes	42 (21.8)	31 (20.8)	

Data are presented as mean and standard deviation (SD) or *n* (%) or median (25th, 75th percentile). ^a^ Independent samples *t*-test; ^b^ Mann−Whitney U test; ^c^ Chi-squared test; ^d^ Chi-squared test with continuity correction; ^e^ Fisher’s exact test. CRF, cancer-related fatigue; NCRF, non-cancer-related fatigue; BMI, body mass index; VAS, visual analogue scale.

**Table 2:** Nutrient intakes of the breast cancer patients with CRF and NCRF.

Variables	NCRF (*n* = 193)	CRF (*n* = 149)	*p*
Energy (kcal/day)	1354.0 (1116.5, 1577.5)	1169.0 (946.0, 1467.0)	<0.001
Protein (g/day)	67.0 (51.5, 88.9)	56.8 (42.3, 72.2)	<0.001
Fat (g/day)	49.4 (36.1, 65.3)	43.5 (30.6, 63.7)	0.018
Carbohydrate (g/day)	151.0 (123.3, 190.9)	133.0 (104.7, 167.3)	<0.001
Dietary fiber (g/day)	9.7 (7.1, 13.3)	7.5 (5.5, 10.0)	<0.001
Cholesterol (mg/day)	587.0 (411.5, 794.5)	551.0 (402.5, 739.5)	0.252
Vitamin A (µgRAE/day)	458.0 (320.5, 637.0)	376.0 (290.5, 514.5)	0.001
Vitamin D (µg/day)	1.5 (0.3, 4.8)	2.0 (1.0, 4.1)	0.602
Vitamin E (mg/day)	16.7 (13.3, 21.4)	14.2 (10.0, 18.1)	<0.001
Vitamin B1 (mg/day)	0.7 (0.5, 0.9)	0.6 (0.4, 0.8)	0.003
Vitamin B2 (mg/day)	1.0 (0.8, 1.4)	0.9 (0.7, 1.2)	0.010
Vitamin B6 (mg/day)	0.2 (0.1, 0.3)	0.1 (0.1, 0.2)	0.001
Vitamin C (mg/day)	116.6 (78.7, 176.5)	89.0 (49.6, 135.7)	<0.001
Folate (µg/day)	109.6 (67.3, 168.4)	95.6 (57.8, 136.3)	0.029
Niacin (mg/day)	13.3 (10.2, 17.0)	10.7 (8.4, 14.7)	<0.001
Calcium (mg/day)	596.0 (386.0, 835.5)	485.0 (295.5, 679.5)	0.002
Phosphorus (mg/day)	937.6 (737.6, 1219.4)	821.5 (616.2, 1023.4)	<0.001
Potassium (mg/day)	1928.3 (1545.0, 2392.9)	1620.7 (1160.1, 2018.1)	<0.001
Sodium (mg/day)	3803.6 (3011.6, 4689.5)	3315.7 (2523.6, 4045.0)	<0.001
Magnesium (mg/day)	281.0 (219.0, 351.0)	227.0 (179.0, 276.5)	<0.001
Iron (mg/day)	17.0 (13.9, 22.3)	14.9 (11.4, 19.2)	<0.001
Iodine (µg/day)	21.5 (13.6, 31.9)	20.7 (14.1, 29.3)	0.211
Zinc (mg/day)	9.9 (7.7, 12.3)	8.4 (6.2, 11.1)	<0.001
Selenium (µg/day)	55.4 (34.9, 78.1)	44.9 (29.3, 66.6)	0.010
Copper (mg/day)	1.7 (1.0, 3.0)	1.1 (0.8, 2.2)	<0.001
Manganese (mg/day)	3.6 (2.7, 4.9)	3.0 (2.1, 4.3)	0.001
Choline (mg/day)	19.1 (11.1, 33.0)	14.4 (4.9, 24.4)	0.001
Biotin (µg/day)	4.0 (1.9, 7.0)	3.0 (1.4, 5.4)	0.011
β-Carotene (µg/day)	1489.4 (793.5, 2790.7)	1254.5 (731.2, 2042.7)	0.113
SFA (g/day)	14.2 (10.7, 19.3)	13.8 (8.6, 19.4)	0.147
MUFA (g/day)	17.4 (12.7, 23.8)	15.8 (10.6, 23.2)	0.101
PUFA (g/day)	8.8 (6.3, 12.5)	7.4 (5.3, 10.1)	0.001
Omega-3 fatty acids (mg/day)	117.5 (77.1, 173.2)	103.2 (61.8, 138.4)	0.007
Omega-6 fatty acids (mg/day)	731.4 (500.4, 1095.5)	603.2 (433.1, 869.7)	0.002
Tryptophan (mg/day)	649.9 (498.3, 892.2)	599.6 (446.8, 746.3)	0.018

Data are presented as median (25th, 75th percentile) for continuous variables. Mann−Whitney U test was used. CRF, cancer-related fatigue; NCRF, non-cancer-related fatigue; SFA, saturated fatty acids; MUFA, monounsaturated fatty acids; PUFA, polyunsaturated fatty acids.

**Table 3:** CHEI component scores of the CRF and NCRF breast cancer patients.

Variables	NCRF (*n* = 193)	CRF (*n* = 149)	*p*
Total CHEI score ^a^	62.0 ± 8.9	58.3 ± 8.4	<0.001
Total grains ^b^	3.4 (2.4, 4.6)	3.5 (2.2, 5.0)	0.567
Whole grains and mixed beans ^b^	0.0 (0.0, 4.0)	0.0 (0.0, 2.8)	0.009
Tubers ^b^	0.0 (0.0, 5.0)	0.0 (0.0, 3.0)	0.262
Total vegetables ^b^	4.1 (2.4, 5.0)	3.5 (2.0, 5.0)	0.062
Dark vegetables ^b^	5.0 (2.8, 5.0)	4.0 (2.2, 5.0)	0.006
Fruits ^b^	10.0 (10.0, 10.0)	10.0 (8.3, 10.0)	0.171
Poultry ^b^	0.0 (0.0, 0.0)	0.0 (0.0, 0.0)	0.813
Red meat ^b^	3.2 (0.5, 5.0)	3.1 (0.1, 5.0)	0.424
Fish and seafood ^b^	5.0 (0.0, 5.0)	5.0 (0.0, 5.0)	0.116
Eggs ^b^	5.0 (2.3, 5.0)	5.0 (5.0, 5.0)	0.215
Dairy ^b^	2.8 (0.0, 5.0)	0.0 (0.0, 5.0)	0.673
Soybeans ^b^	0.0 (0.0, 4.2)	0.0 (0.0, 0.0)	0.022
Seeds and nuts ^b^	0.0 (0.0, 0.0)	0.0 (0.0, 0.0)	0.020
Cooking oils ^b^	10.0 (10.0, 10.0)	10.0 (10.0, 10.0)	0.421
Sodium ^b^	3.0 (0.8, 5.3)	3.1 (1.1, 4.9)	0.804
Alcohol ^b^	5.0 (5.0, 5.0)	5.0 (5.0, 5.0)	1.000
Added sugars ^b^	5.0 (5.0, 5.0)	5.0 (5.0, 5.0)	1.000

Data are presented as mean ± SD or median (25th, 75th percentile). ^a^ Independent samples *t*-test; ^b^ Mann−Whitney U test. CHEI, Chinese Healthy Eating Index; CRF, cancer-related fatigue; NCRF, non-cancer-related fatigue.

**Table 4:** Multivariate logistic regression analysis of nutrient factors influencing the occurrence of CRF in breast cancer patients (*n* = 342).

Variables	OR	95% CI	*p*
Protein	0.98	0.97, 0.99	0.016
Fat	1.01	0.99, 1.03	0.199
Carbohydrate	0.99	0.99, 1.01	0.831
Dietary fiber	0.94	0.88, 0.99	0.024
Vitamin A	0.99	0.99, 1.00	0.006
Vitamin E	0.94	0.90, 0.98	0.003
Vitamin B1	1.26	0.35, 4.54	0.727
Vitamin B2	0.79	0.54, 1.16	0.231
Vitamin B6	0.23	0.05, 1.13	0.070
Vitamin C	0.99	0.99, 1.00	0.113
Folate	0.99	0.99, 1.00	0.701
Niacin	0.96	0.91, 1.01	0.147
Calcium	0.99	0.99, 1.00	0.153
Phosphorus	0.99	0.99, 1.00	0.021
Potassium	0.99	0.99, 1.00	0.018
Sodium	0.99	0.99, 1.00	0.124
Magnesium	0.99	0.99, 1.00	0.008
Iron	0.95	0.90, 0.99	0.039
Zinc	0.95	0.87, 1.03	0.183
Selenium	0.99	0.99, 1.00	0.089
Copper	0.75	0.60, 0.95	0.018
Manganese	0.99	0.94, 1.06	0.933
Choline	1.00	0.99, 1.00	0.973
Biotin	0.99	0.98, 1.02	0.728
PUFA	0.96	0.89, 1.02	0.197
Omega-3 fatty acids	0.99	0.99, 1.00	0.498
Omega-6 fatty acids	0.99	0.99, 1.00	0.240
Tryptophan	0.99	0.99, 1.00	0.654

Multiple logistic regression was used after adjusting for age, BMI, VAS score, family monthly income, sleep disorders, anxiety, depression, and energy. OR, odds ratio; CI, confidence interval; PUFA, polyunsaturated fatty acids.

**Table 5:** Multiple logistic regression analysis of CHEI and its components influencing the occurrence of CRF in breast cancer patients (*n* = 342).

Variables	OR	95% CI	*p*
Total CHEI score	0.95	0.93, 0.98	0.002
Whole grains and mixed beans	0.85	0.75, 0.96	0.010
Dark vegetables	0.88	0.76, 1.02	0.092
Soybeans	0.90	0.79, 1.01	0.081
Seeds and nuts	0.89	0.76, 1.04	0.153

Multiple logistic regression was used after adjusting for age, BMI, VAS score, family monthly income, sleep disorders, anxiety, and depression. OR, odds ratio; CI, confidence interval; CHEI, Chinese Healthy Eating Index.

**Table 6:** Plasma TRP and KYN levels of breast cancer patients with CRF and NCRF.

Variables	NCRF (*n* = 72)	CRF (*n* = 34)	*p*
TRP (μmol/L)	24.77 (23.38, 27.42)	23.14 (19.18, 25.20)	0.001
KYN (μmol/L)	7.54 (6.51, 8.30)	7.04 (6.54, 8.44)	0.488
KYN/TRP	0.30 (0.26, 0.33)	0.35 (0.29, 0.38)	<0.001

Data are shown as median (25th, 75th percentile). Mann−Whitney U test was used. CRF, cancer-related fatigue; NCRF, non-cancer-related fatigue; TRP, tryptophan; KYN, kynurenine; KYN/TRP, kynurenine/tryptophan ratio.

**Table 7:** Mediating effect of gut microbiota on the association between diet and the occurrence of CRF in breast cancer patients (*n* = 64).

Variables	ATE			ADE			ACME		
	Estimate	95% CI	*p*	Estimate	95% CI	*p*	Estimate	95% CI	*p*
Total CHEI score ^a^									
Sobs index	−0.0007	−0.0047, −0.0001	0.010	−0.0001	−0.0023, 0.0014	0.100	−0.0005	−0.0051, −0.0001	0.034
Chao index	−0.0007	−0.0047, −0.0001	0.013	−0.0002	−0.0023, 0.0013	0.090	−0.0005	−0.0050, −0.0001	0.033
Shannon index	−0.0006	−0.0047, −0.0001	0.034	−0.0003	−0.0053, 0.0019	0.107	−0.0002	−0.0048, 0.0022	0.296
PC1	−0.0012	−0.0057, −0.0001	0.032	−0.0003	−0.0037, 0.0042	0.171	−0.0009	−0.0068, 0.0001	0.086
Bacteroidota	−0.0007	−0.0050, −0.0001	0.037	−0.0002	−0.1847, 1.2703	0.112	−0.0005	−0.0052, 0.0001	0.086
unclassified_k_norank_d_Bacteria	−0.0008	−0.0051, −0.0001	0.032	−0.0005	−0.0054, 0.0018	0.088	−0.0003	−0.0051, 0.0019	0.448
Vitamin A ^b^									
Sobs index	−0.0004	−0.0007, 0.0001	0.058	−0.0003	−0.0006, 0.0001	0.118	−0.0001	−0.0003, 0.0001	0.226
Shannon index	−0.0003	−0.0007, 0.0001	0.084	−0.0003	−0.0006, 0.0001	0.121	−0.0001	−0.0003, 0.0001	0.376
Desulfobacterota	−0.0004	−0.0007, 0.0001	0.052	−0.0002	−0.0006, 0.0001	0.106	−0.0001	−0.0004, 0.0002	0.322
Phosphorus ^b^									
Desulfobacterota	−0.0002	−0.0005, 0.0002	0.140	−0.0001	−0.0005, 0.0001	0.140	−0.0001	−0.0003, 0.0003	0.400
Copper ^b^									
Proteobacteria	−0.1274	−0.2408, 0.0068	0.064	−0.1079	−0.2336, 0.0228	0.117	−0.0196	−0.0779, 0.0389	0.476

^a^ The mediating effect of gut microbiota on total CHEI score and CRF in patients with breast cancer after adjustment for age, BMI, VAS score, family monthly income, sleep disorders, anxiety, and depression. ^b^ The mediating effect of gut microbiota on dietary intake and CRF in patients with breast cancer after adjustment for age, BMI, VAS score, family monthly income, sleep disorders, anxiety, depression, and energy. The 95% CI was computed from 5000 nonparametric bootstrap replicates. PC1 presented value is PC1 × 100; Bacteroidota and Proteobacteria are scaled up by a factor of 1000 for calculations; unclassified_k__norank_d__Bacteria and Desulfobacterota are scaled up by a factor of 100,000 for calculations. ACME, average causal mediation effect; ADE, average direct effect; ATE, average total effect; CHEI, Chinese Healthy Eating Index; CI, confidence interval; CRF, cancer-related fatigue.

## Data Availability

The raw sequence data reported in this paper have been deposited in the Genome Sequence Archive [57] in the National Genomics Data Center [58], China National Center for Bioinformation/Beijing Institute of Genomics, Chinese Academy of Sciences (GSA: CRA020711) (publicly accessible at https://ngdc.cncb.ac.cn/gsa on 26 November 2026).

## Supplementary Materials

The following supporting information can be downloaded at: https://www.mdpi.com/article/10.3390/nu16244371/s1, Table S1: The International Classification of Diseases, 10th Revision (ICD-10), criteria for cancer-related fatigue; Table S2: Diagnostic interview guide for cancer-related fatigue; Table S3: Association between gut microbiota and CRF in breast cancer patients (*n* = 64); Table S4: Association between dietary intake and gut microbiota in breast cancer patients (*n* = 64).
