# Supplementary material for: The Mediating Role of Gut Microbiota on the Association Between Dietary Quality and Cancer-Related Fatigue Among Breast Cancer Patients: A Cross-Sectional Study
Source: Nutrients. 2024 Dec 18;16(24):4371. doi: 10.3390/nu16244371 (PMC11676191; doi:10.3390/nu16244371)
Supplement: Supplementary file 1 [file nutrients-16-04371-s001.zip › nutrients-3366762-supplementary.pdf]

Supplementary Materials:

Methods used to assess participants' sleep disorder, pain, anxiety and depression status, and physical activity:

The Pittsburgh Sleep Quality Index (PSQI) was used to assess sleep disorder in patients, where a PSQI score of < 8 indicated normal, whereas a score of ≥ 8 indicated poor sleep quality. Anxiety and depression status were evaluated using the self-reported Hospital Anxiety and Depression Scale (HADS), which consists of an anxiety subscale (7 items, HADS-A) and a depression subscale (7 items, HADS-D). Visual Analogue Scale (VAS) was used to for assessing pain. Physical activity level was assessed by the International Physical Activity Questionnaire Short Form (IPAQ-SF) to calculate the metabolic equivalent (MET) and then classified into three levels: low (< 600 MET/min/week), moderate (600 to 1500 MET/min/week), and high (> 1500 MET/min/week).

Table S1. The International Classification of Diseases 10th Revision (ICD-10) criteria for cancer-related fatigue.

six (or more) of the following symptoms have been present every day or nearly every day during the same 2-week period in the past month, and at least one of the symptoms is (A1) significant fatigue.	
A1	Significant fatigue, diminished energy, or increased need to rest, disproportionate to any recent change in activity level
A2	complaints of generalized weakness or limb heaviness
A3	diminished concentration or attention
A4	decreased motivation or interest in engaging in usual activities
A5	insomnia or hypersomnia
A6	experience of sleep as unrefreshing or nonrestorative
A7	perceived need to struggle to overcome inactivity
A8	marked emotional reactivity (e.g. sadness, frustration or irritability) to feeling fatigued
A9	difficulty completing daily tasks attributed to feeling fatigue
A10	perceived problems with short-term memory
A11	post-exertional malaise lasting several hours
B	The symptoms cause clinically significant distress or impairment in social, occupational, or other important areas of functioning
C	There is evidence from the history, physical examination, or laboratory findings that the symptoms are a consequence of cancer or cancer therapy
D	The symptoms are not primarily a consequence of comorbid psychiatric disorders such as major depression, somatization disorder, somatoform disorder, or delirium

Table S2. Diagnostic interview guide for cancer-related fatigue.

**Diagnostic Interview Guide for Cancer-Related Fatigue**			
**NOTE: Capitalized text represents instructions to the interviewer. Text in quotations represents statements to be read verbatim to respondent.**			
1. "Over the past month, has there been at least a 2-week period when you had significant fatigue, a lack of energy, or an increased need to rest every day or nearly every day? "	CIRCLE ONE:	**YES**	**NO**
**IF NO, STOP HERE. IF YES, CONTINUE**			
"For each of the following questions, focus on the worst 2 weeks in the past month (or else the past 2 weeks if you felt equally fatigued for the entire month). "			
2. "Did you feel weak all over or heavy all over? (every day or nearly every day?) "	CIRCLE ONE:	**YES**	**NO**
3. "Did you have trouble concentrating or paying attention? (every day or nearly every day?) "	CIRCLE ONE:	**YES**	**NO**
4. "What about losing your interest or desire to the things you usually do? (every day or nearly every day?) "	CIRCLE ONE:	**YES**	**NO**
5. "How were you sleeping? Did you have trouble falling asleep, staying asleep, or waking too early? Or did you find yourself sleeping too much compared to what you usually sleep? (every day or nearly every day?) "	CIRCLE ONE:	**YES**	**NO**
6. "Have you found that you usually don't feel rested or refreshed after you have slept? (every day or nearly every day?) "	CIRCLE ONE:	**YES**	**NO**
7. "Did you have struggle or push yourself to do anything? (every day or nearly every day?) "	CIRCLE ONE:	**YES**	**NO**
8. "Did you find yourself feeling sad, frustrated, or irritable because you felt fatigued? (every day or nearly every day?)"	CIRCLE ONE:	**YES**	**NO**
9. "Did you have difficulty finishing something you had started to do because of feeling fatigued? (every day or nearly every day?) "	CIRCLE ONE:	**YES**	**NO**
10. "Did you have trouble remembering things? For example, did you have trouble remembering where your keys were or what someone had told you a little while ago? (every day or nearly every day?)"	CIRCLE ONE:	**YES**	**NO**
11. "Did you find yourself feeling sick or unwell for several hours after you had done something that took some effort (every time or nearly every time?)"	CIRCLE ONE:	**YES**	**NO**
**IF LESS THAN SIX ITEMS INCLUDING #1 ARE MARKED YES, STOP HERE**			
12. "Has fatigue made it hard for you to do your work, take care of things at home, or get along with other people?"	CIRCLE ONE:	**YES**	**NO**
**IF # 12 IS NO, STOP HERE**			
13. Is there evidence from the history, physical examination, or laboratory findings that the symptoms are a consequence	CIRCLE ONE:	**YES**	**NO**

of cancer or cancer treatment?

IF # 13 IS NO, STOP HERE

14. Are the symptoms a consequence of co-morbid psychiatric disorders such as major depression, somatization disorder, somatoform disorder, or delirium? CIRCLE ONE: **YES NO**

IF # 14 IS YES, PATIENT DOES NOT MEET CRITERIA FOR CANCER-RELATED FATIGUE

IF # 14 IS NO, PATIENT MEETS CRITERIA FOR CANCER-RELATED FATIGUE

Table S3 – Association between gut microbiota and CRF in breast cancer patients (n = 64).

Variables	OR	95% CI	*p*
Sobs index	0.99	0.98, 0.99	0.002
Simpson index [a]	1.05	0.98, 1.12	0.170
Chao index	0.99	0.98, 0.99	0.002
Shannon index	0.11	0.02, 0.65	0.015
PC1 [a]	0.90	0.84, 0.96	0.002
p__Firmicutes [b]	0.99	0.99, 1.00	0.537
p__Actinobacteriota [b]	1.01	1.00, 1.02	0.013
p__Synergistota [c]	0.96	0.91, 1.02	0.237
p__Bacteroidota [b]	0.98	0.97, 0.99	0.004
p__Cyanobacteria [c]	1.00	0.99, 1.02	0.728
p__Proteobacteria [b]	1.01	1.00, 1.01	0.042
p__Fusobacteriota [c]	0.99	0.99, 1.00	0.447
p__Verrucomicrobiota [c]	0.99	0.99, 1.00	0.142
p__Desulfobacterota [c]	0.99	0.98, 0.99	0.013
p__unclassified_k__norank_d__Bacteria [c]	0.98	0.97, 0.99	0.035
p__Campilobacterota [c]	0.89	0.74, 1.08	0.231
p__Patescibacteria [c]	1.03	0.99, 1.07	0.061
g__Blautia [b]	1.00	0.99, 1.01	0.707
g__Faecalibacterium [c]	0.99	0.99, 1.00	0.420
g__Escherichia-Shigella [b]	1.01	0.99, 1.01	0.181
g__Bacteroides [b]	0.99	0.98, 0.99	0.013
g__Bifidobacterium [b]	1.01	1.00, 1.02	0.010
g__Streptococcus [b]	0.99	0.99, 1.01	0.953
g__Prevotella [c]	0.99	0.99, 1.00	0.132
g__Agathobacter [b]	0.99	0.98, 1.02	0.693
g__Ruminococcus_gnavus_group [b]	1.01	0.99, 1.02	0.253
g__unclassified_f__Lachnospiraceae [b]	0.99	0.97, 1.03	0.847
g__Ruminococcus [c]	1.00	0.99, 1.00	0.544
g__Eubacterium_hallii_group [b]	1.02	0.99, 1.06	0.210
g__Fusicatenibacter [b]	1.01	0.98, 1.03	0.699
g__Ruminococcus_torques_group [b]	0.99	0.97, 1.03	0.800
g__Anaerostipes [c]	1.00	0.99, 1.00	0.065

Multiple logistic regression was used after adjusting for age, BMI, VAS score, family monthly income, sleep disorder, anxiety, depression and energy.

[a] represent original data has been scaled up by a factor of 100 to calculate.

[b] represent original data has been scaled up by a factor of 1000 to calculate.

[c] represent original data has been scaled up by a factor of 100000 to calculate.

CI, confidence interval; CRF, cancer-related fatigue; OR, odds ratio.

Table S4. Association between dietary intake and gut microbiota in breast cancer patients (n = 64).

	R^2	β	95% CI	*p*
		Sobs index		
Total CHEI score [a]	0.20	0.39	2.12, 11.08	0.005
Whole grains and mixed beans [a]	0.09	0.16	−6.53, 25.88	0.236
Protein [b]	0.10	0.15	−0.10, 2.28	0.432
Dietary fiber [b]	0.09	0.11	−6.47, 12.73	0.516
Vitamin A [b]	0.15	0.28	0.01, 0.17	0.047
Vitamin E [b]	0.11	0.19	−2.12, 8.16	0.246
Phosphorus [b]	0.09	0.14	−0.09, 0.19	0.475
Potassium [b]	0.10	0.14	−0.04, 0.10	0.444
Magnesium [b]	0.10	0.16	−0.27, 0.73	0.365
Iron [b]	0.12	0.23	−1.26, 8.63	0.141
Copper [b]	0.14	0.28	−2.40, 61.06	0.069
		Chao index		
Total CHEI score [a]	0.19	0.37	2.03, 12.55	0.007
Whole grains and mixed beans [a]	0.10	0.17	−7.23, 30.52	0.222
Protein [b]	0.12	0.15	−1.15, 2.65	0.430
Dietary fiber [b]	0.12	0.13	−6.87, 15.37	0.447
Vitamin A [b]	0.16	0.26	−0.01, 0.19	0.071
Vitamin E [b]	0.13	0.19	−2.47, 9.47	0.245
Phosphorus [b]	0.11	0.12	−0.11, 0.21	0.523
Potassium [b]	0.12	0.14	−0.05, 0.11	0.435
Magnesium [b]	0.12	0.15	−0.33, 0.84	0.391
Iron [b]	0.13	0.20	−2.11, 9.43	0.209
Copper [b]	0.15	0.25	−5.66, 68.35	0.095
		Shannon index		
Total CHEI score [a]	0.41	0.42	0.02, 0.06	0.001
Whole grains and mixed beans [a]	0.29	0.16	−0.03, 0.12	0.204
Protein [b]	0.28	−0.14	−0.01, 0.01	0.412
Dietary fiber [b]	0.28	0.14	−0.03, 0.07	0.377
Vitamin A [b]	0.33	0.27	0.00, 0.01	0.033
Vitamin E [b]	0.27	−0.06	−0.03, 0.02	0.698
Phosphorus [b]	0.27	−0.07	−0.01, 0.01	0.667
Potassium [b]	0.27	0.07	0.00, 0.00	0.676
Magnesium [b]	0.29	0.18	−0.01, 0.01	0.257
Iron [b]	0.27	0.06	−0.02, 0.03	0.656
Copper [b]	0.32	0.26	−0.01, 0.30	0.053
		PC1		
Total CHEI score [a]	0.29	0.27	0.07, 1.50	0.033
Whole grains and mixed beans [a]	0.25	−0.13	−3.79, 1.25	0.316
Protein [b]	0.24	−0.05	−0.30, 0.22	0.758
Dietary fiber [b]	0.24	0.03	−1.33, 1.66	0.827

Vitamin A [b]	0.25	0.05	−0.01, 0.02	0.683
Vitamin E [b]	0.25	−0.05	−0.95, 0.66	0.720
Phosphorus [b]	0.24	−0.06	−0.03, 0.02	0.735
Potassium [b]	0.24	−0.01	−0.01, 0.01	0.979
Magnesium [b]	0.25	0.09	−0.06, 0.10	0.599
Iron [b]	0.24	0.04	−0.68, 0.89	0.791
Copper [b]	0.28	0.23	−0.73, 9.18	0.093
	Actinobacteriota			
Total CHEI score [a]	0.19	−0.25	−8.29, 0.27	0.066
Whole grains and mixed beans [a]	0.13	−0.02	−16.03, 14.04	0.895
Protein [b]	0.14	−0.10	−1.93, 1.09	0.582
Dietary fiber [b]	0.21	0.28	−0.02, 0.18	0.095
Vitamin A [b]	0.15	−0.13	−0.12, 0.04	0.347
Vitamin E [b]	0.14	−0.01	−4.99, 4.63	0.941
Phosphorus [b]	0.15	−0.17	−0.19, 0.07	0.349
Potassium [b]	0.16	−0.19	−0.10, 0.03	0.288
Magnesium [b]	0.18	−0.28	−0.83, 0.08	0.106
Iron [b]	0.14	0.09	−3.24, 6.05	0.546
Copper [b]	0.18	−0.25	−54.34, 4.51	0.095
	Bacteroidota			
Total CHEI score [a]	0.23	0.34	1.66, 12.45	0.011
Whole grains and mixed beans [a]	0.14	−0.11	−27.49, 11.23	0.404
Protein [b]	0.13	0.03	−1.80, 2.14	0.863
Dietary fiber [b]	0.13	0.05	−9.85, 13.25	0.769
Vitamin A [b]	0.19	0.27	−0.01, 0.20	0.052
Vitamin E [b]	0.13	0.02	−5.92, 6.58	0.917
Phosphorus [b]	0.14	0.12	−0.11, 0.22	0.509
Potassium [b]	0.14	0.15	−0.05, 0.12	0.396
Magnesium [b]	0.16	0.23	−0.20, 1.00	0.191
Iron [b]	0.15	0.16	−2.98, 9.02	0.317
Copper [b]	0.18	0.25	−5.30, 71.11	0.090
	Proteobacteria			
Total CHEI score [a]	0.27	−0.23	−12.23, 0.57	0.073
Whole grains and mixed beans [a]	0.24	−0.11	−31.96, 12.60	0.388
Protein [b]	0.30	0.01	−2.14, 2.18	0.982
Dietary fiber [b]	0.30	−0.06	−15.30, 10.00	0.676
Vitamin A [b]	0.32	−0.15	−0.18, 0.05	0.238
Vitamin E [b]	0.30	−0.02	−7.29, 6.41	0.898
Phosphorus [b]	0.30	0.01	−0.18, 0.19	0.958
Potassium [b]	0.30	−0.05	−0.11, 0.08	0.758
Magnesium [b]	0.31	−0.12	−0.92, 0.40	0.434
Iron [b]	0.32	−0.19	−11.10, 1.93	0.164

Copper [b]	0.35	−0.26	−83.43, −0.52	0.047
	Desulfobacterota			
Total CHEI score [a]	0.15	0.19	−3.59, 19.02	0.177
Whole grains and mixed beans [a]	0.14	0.12	−21.66, 56.02	0.379
Protein [b]	0.19	0.27	−0.83, 6.76	0.123
Dietary fiber [b]	0.19	0.22	−7.02, 37.72	0.174
Vitamin A [b]	0.26	0.36	0.07, 0.45	0.008
Vitamin E [b]	0.18	0.19	−4.65, 19.62	0.221
Phosphorus [b]	0.23	0.40	0.05, 0.67	0.025
Potassium [b]	0.18	0.21	−0.07, 0.26	0.236
Magnesium [b]	0.17	0.14	−0.71, 1.68	0.419
Iron [b]	0.18	0.19	−4.52, 19.00	0.222
Copper [b]	0.18	0.17	−30.81, 121.84	0.237
	unclassified_k__norank_d__Bacteria			
Total CHEI score [a]	0.23	0.43	1.43, 5.82	0.002
Whole grains and mixed beans [a]	0.09	−0.14	−12.37, 3.85	0.297
Protein [b]	0.08	0.07	−0.67, 0.98	0.708
Dietary fiber [b]	0.08	0.01	−4.77, 4.94	0.972
Vitamin A [b]	0.09	0.15	−0.02, 0.07	0.307
Vitamin E [b]	0.08	0.05	−2.26, 2.99	0.782
Phosphorus [b]	0.08	0.11	−0.05, 0.09	0.571
Potassium [b]	0.08	0.05	−0.03, 0.04	0.801
Magnesium [b]	0.10	0.20	−0.11, 0.40	0.261
Iron [b]	0.10	0.20	−0.95, 4.07	0.217
Copper [b]	0.09	0.15	−8.55, 24.15	0.343
	Bacteroides			
Total CHEI score [a]	0.11	0.27	−0.19, 8.69	0.060
Whole grains and mixed beans [a]	0.05	−0.08	−19.72, 11.40	0.594
Protein [b]	0.06	0.12	−1.09, 2.05	0.543
Dietary fiber [b]	0.06	0.11	−6.38, 12.06	0.540
Vitamin A [b]	0.07	0.17	−0.04, 0.13	0.257
Vitamin E [b]	0.05	0.01	−4.98, 5.02	0.994
Phosphorus [b]	0.06	0.17	−0.08, 0.19	0.393
Potassium [b]	0.08	0.24	−0.02, 0.11	0.208
Magnesium [b]	0.08	0.237	−0.18, 0.79	0.207
Iron [b]	0.09	0.23	−1.34, 8.17	0.156
Copper [b]	0.08	0.20	−10.69, 51.20	0.195
	Bifidobacterium			
Total CHEI score [a]	0.19	−0.26	−7.80, 0.13	0.058
Whole grains and mixed beans [a]	0.14	−0.05	−16.70, 11.15	0.691
Protein [b]	0.15	−0.11	−1.81, 0.98	0.557
Dietary fiber [b]	0.21	−0.32	−15.78, 0.07	0.052

Vitamin A [b]	0.17	−0.15	−0.11, 0.03	0.283
Vitamin E [b]	0.15	0.01	−4.26, 4.62	0.934
Phosphorus [b]	0.17	−0.19	−0.18, 0.06	0.296
Potassium [b]	0.17	−0.20	−0.09, 0.02	0.248
Magnesium [b]	0.19	−0.29	−0.78, 0.07	0.097
Iron [b]	0.16	0.10	−2.87, 5.70	0.512
Copper [b]	0.19	−0.24	−49.56, 4.88	0.106

[a] Multiple linear regression was used after adjusting for age, BMI, VAS score, family monthly income, sleep disorder, anxiety and depression.

[b] Multiple linear regression was used after adjusting for age, BMI, VAS score, family monthly income, sleep disorder, anxiety, depression and energy.

PC1 presented value is PC1 × 100; Actinobacteriota, Bacteroidota, Proteobacteria, *Bacteroides* and *Bifidobacterium* are scaled up by a factor of 1000 to calculate; unclassified_k__norank_d__Bacteria and Desulfobacterota are scaled up by a factor of 100000 to calculate.
